# Multisite tyrosine phosphorylation of the N‐terminus of Mint1/X11α by Src kinase regulates the trafficking of amyloid precursor protein

**DOI:** 10.1111/jnc.13571

**Published:** 2016-03-01

**Authors:** Christopher J. R. Dunning, Hannah L. Black, Katie L. Andrews, Elizabeth C. Davenport, Michael Conboy, Sangeeta Chawla, Adam A. Dowle, David Ashford, Jerry R. Thomas, Gareth J. O. Evans

**Affiliations:** ^1^ Department of BiologyUniversity of YorkYorkUK; ^2^ Present address: Neuronal Survival UnitDepartment of Experimental Medical ScienceWallenberg Neuroscience CenterLund UniversityBMC B11 221 84LundSweden

**Keywords:** amyloid precursor protein, intracellular trafficking, Mint1, protein phosphorylation, Src, tyrosine kinase

## Abstract

Mint/X11 is one of the four neuronal trafficking adaptors that interact with amyloid precursor protein (APP) and are linked with its cleavage to generate β‐amyloid peptide, a key player in the pathology of Alzheimer's disease. How APP switches between adaptors at different stages of the secretory pathway is poorly understood. Here, we show that tyrosine phosphorylation of Mint1 regulates the destination of APP. A canonical SH2‐binding motif (^202^ YEEI) was identified in the N‐terminus of Mint1 that is phosphorylated on tyrosine by C‐Src and recruits the active kinase for sequential phosphorylation of further tyrosines (Y191 and Y187). A single Y202F mutation in the Mint1 N‐terminus inhibits C‐Src binding and tyrosine phosphorylation. Previous studies observed that co‐expression of wild‐type Mint1 and APP causes accumulation of APP in the trans‐Golgi. Unphosphorylatable Mint1 (Y202F) or pharmacological inhibition of Src reduced the accumulation of APP in the trans‐Golgi of heterologous cells. A similar result was observed in cultured rat hippocampal neurons where Mint1(Y202F) permitted the trafficking of APP to more distal neurites than the wild‐type protein. These data underline the importance of the tyrosine phosphorylation of Mint1 as a critical switch for determining the destination of APP.

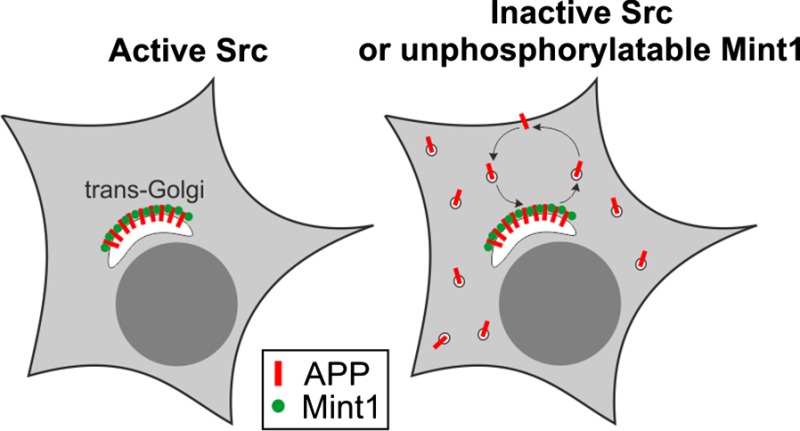

The regulation of amyloid precursor protein (APP) trafficking is poorly understood. We have discovered that the APP adapter, Mint1, is phosphorylated by C‐Src kinase. Mint1 causes APP accumulation in the trans‐Golgi network, whereas inhibition of Src or mutation of Mint1‐Y202 permits APP recycling. The phosphorylation status of Mint1 could impact on the pathological trafficking of APP in Alzheimer's disease.

**Abbreviations used**
AD: Alzheimer's disease
APLP: APP‐like protein
APP: amyloid precursor protein
PTB: phosphotyrosine binding

Alzheimer's disease (AD) is caused by the toxicity of soluble β‐amyloid oligomers that arise from the cleavage of amyloid precursor protein (APP) by secretase enzymes (Shankar and Walsh 2009). APP is ubiquitous in the body and although its exact function is unclear, the consensus is that APP is a receptor and/or cell adhesion molecule (Zheng and Koo 2011). APP and APP‐like protein knockout and knock‐in experiments in various model organisms have implicated the protein in neuronal development and synaptic function, including plasticity (Muller *et al*. 1994; Seabrook *et al*. 1999; Merdes *et al*. 2004). However, the fact that β‐amyloid accumulation is mainly restricted to the brain in AD suggests it is because of neuronal‐specific interactors and/or the demands of trafficking APP long distances from the neuronal cell soma to distal synapses.

APP trafficking and processing is coordinated by several multi‐domain adapter proteins, including Dab1&2, Fe65, JIP1 and Mint1,2,3/X11α,β,γ, that interact with the C‐terminus of APP via phosphotyrosine‐binding (PTB) domains and link APP to a host of factors, including sorting, signalling and motor proteins (Kawasumi *et al*. 2004; King and Scott Turner 2004). Of these, Dab1, Fe65 and Mint1 and 2 are neuron specific. Because the proper processing of APP relies on its co‐localisation with the correct secretase enzymes, disruption of APP–adapter interactions often affects APP trafficking and proteolysis, leading to increased production of β‐amyloid peptide (Sastre *et al*. 1998; Lau *et al*. 2000; Mueller *et al*. 2000; Ando *et al*. 2001; Ho *et al*. 2002, 2008; Hoe *et al*. 2008; Kondo *et al*. 2010). It is hypothesised that each adapter is responsible for a specific step in the trafficking and processing of APP, but how spatial and temporal specificity in APP trafficking is achieved is poorly understood. Phosphorylation certainly plays a role in this process because APP can be phosphorylated on tyrosine and threonine residues in its ‘YENPTY’ motif at the C‐terminus, which interacts with PTB domain‐ or SH2 domain‐containing adapters and the μ subunit of AP‐4 (Tarr *et al*. 2002; Burgos *et al*. 2010). Indeed, Fe65, Mint2 and JIP1 have differential affinity for phosphorylated and unphosphorylated APP, however binding of Dab1 and Mint1 to APP is unaffected by the phosphorylation status of APP (Borg *et al*. 1996; Ando *et al*. 2001; Tamayev *et al*. 2009), suggesting alternative mechanisms might exist for their regulation.

In a search for a function of the N‐terminus of Mint1 we identified a canonical SH2 ligand (Y^202^EEI) that is phosphorylated by C‐Src and mediates recruitment of the kinase and the sequential phosphorylation of two adjacent Mint1 tyrosine residues. Stabilisation of APP metabolism by Mint1 is abolished by mutation of the C‐Src phosphorylation site or inhibition of Src, thus revealing a regulatory role for the Mint1 N‐terminus in interactions with APP. Furthermore, trafficking of APP to distal synapses in neurons is also dependent on Mint‐Y202. We propose that the phosphorylation status of Mint1‐Y202 acts as a switch to regulate the destination of APP.

## Materials and methods

### Materials

Anti‐phosphotyrosine mouse monoclonal antibody (clone PY20) was purchased from BD Bioscience (Oxford, UK). Rabbit monoclonal anti‐myc and rabbit polyclonal anti‐FLAG and anti‐Src pY416 were from Cell Signaling Technologies (Hitchin, UK). Rat monoclonal anti‐HA (clone 3F10) was from Roche (Welwyn Garden City, UK). Rabbit anti‐TGN46 was a gift from Dr Daniel Ungar (University of York). Alexa‐fluor‐conjugated secondary antibodies were from Invitrogen (Paisley, UK). The Src family kinase inhibitor, PP2, was obtained from Tocris‐Cookson (Bristol, UK). Rabbit polyclonal anti‐GFP (green fluorescent protein) was from Insight Biotechnology (Wembley, UK). Affinity‐purified anti‐Mint‐PY202 rabbit polyclonal antibody, raised to the peptide CGLQEHVpYEEIGDA, was custom made by Genscript (Piscataway, NJ, USA). Unless stated otherwise, all other reagents were from Sigma (Poole, UK).

### Plasmids

**Figure 1:**
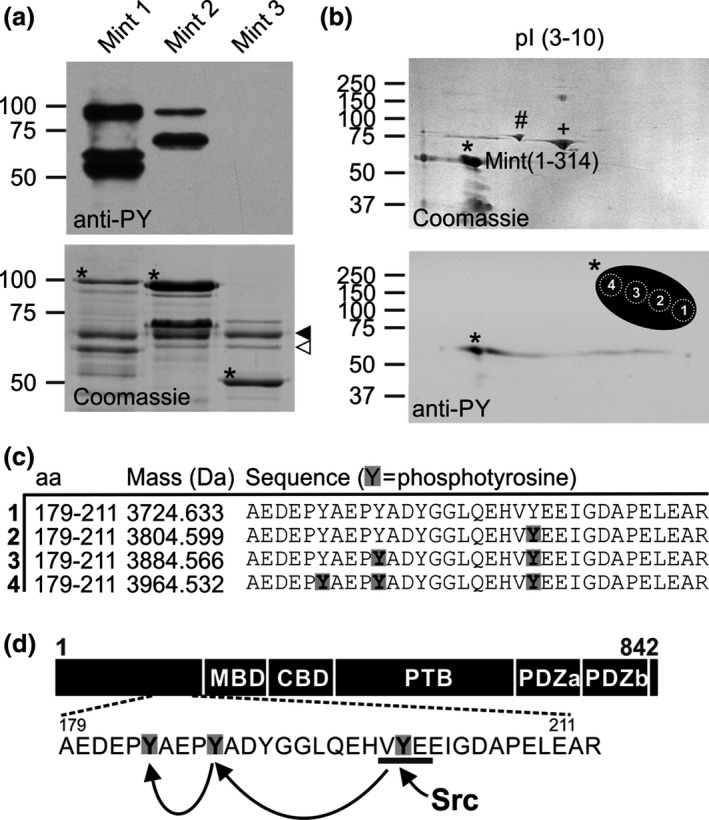
The Mint1 N‐terminus is phosphorylated sequentially by C‐Src. (a) Recombinant GST fusions of the N‐terminal domains of Mint1(1‐314), Mint2(1‐341) and Mint3(1‐184) were subjected to an *in vitro* kinase assay with C‐Src. Tyrosine phosphorylation was detected by immunoblotting (top panel) and protein loading was confirmed by Coomassie staining [bottom panel: asterisks indicate Mint proteins; solid arrowhead = bovine serum albumin (BSA); hollow arrowhead = C‐Src]. Lower molecular weight proteolytic fragments of Mints 1 and 2 were also phosphorylated. (b) Twenty micrograms of phosphorylated Mint1(1‐314) was separated by 2D gel electrophoresis and stained with Coomassie (asterisk, top panel; + = BSA and # = dnaK). Phosphorylation was confirmed by immunoblotting (asterisk, bottom panel). A schematic of the Mint1 spot indicates how it was sampled at four sites by mass spectrometry. (c) Sequences and masses of the predominant phosphopeptides identified in spots 1–4. Phosphorylated tyrosine residues are highlighted in grey. (d) Schematic of the domain structure of Mint1 indicating the location of the three tyrosine residues sequentially phosphorylated by C‐Src.

All Mint1 plasmids were prepared by PCR from a mouse cDNA clone (IMAGE ID 9055793) obtained from GeneService (Cambridge, UK). Mammalian epitope‐tagged Mint1 expression plasmids comprised pMH‐Mint(1‐314)‐HA and pMH‐myc‐Mint1(1‐431), pMH‐myc‐Mint1(1‐842) and pmCerulean‐N1‐Mint(1‐842). A plasmid encoding human APP(1‐751)‐FLAG in a pEGFP‐N1 backbone was prepared using IMAGE clone 6152423. Bacterial expression plasmids included pGEX‐6P1‐Mint1(1‐314), pGEX‐6P1‐Mint1(1‐431), pGEX‐6P1‐Mint3(1‐180) and pQE30‐myc‐Mint(1‐842). The Mint1‐Y202F mutation was introduced into the relevant Mint1 plasmids using the Quikchange system (Stratagene, Stockport, UK) according to the manufacturer's instructions. pGEX2T‐Mint2(1‐341) and pQE30‐Munc18 were generous gifts from the laboratory of Prof Bob Burgoyne, University of Liverpool, UK. Plasmids for expressing recombinant active His‐C‐Src in *Escherichia coli*, pGEX4T‐1‐PTP1B‐3C‐His‐Src(Δ80), and expressing C‐Src‐FLAG in mammalian cells have been described elsewhere (Keenan *et al*. 2015). It should be noted that all bacterial and mammalian expressed Mint1 proteins that contain the N‐terminus had higher than predicted molecular masses when separated by sodium dodecyl sulphate–polyacrylamide gel electrophoresis (SDS‐PAGE). For example, GST‐Mint(1‐431), predicted *M*_r_ = 72 kDa, had an apparent *M*_r_ of ~ 120 kDa (Fig. 1b). We assume the discrepancy is because of the nature of the primary structure of the Mint1 N‐terminus.

### Cell culture and transfection

Flp‐in T‐REx HeLa cells stably expressing Tet‐inducible APP‐FLAG and COS‐7 cells were cultured in Dulbecco's modified Eagle's medium supplemented with 10% foetal calf serum and penicillin/streptomycin (Invitrogen). Cells were transfected 24 h after plating with EcoTransfect transfection reagent according to the manufacturer's instructions (Oz Biosciences, Nottingham, UK). Hippocampal and cortical neurons were prepared from newborn Wistar rat pups of either sex (Harlan) as described previously (Belfield *et al*. 2006) and approved by the Biology Ethics Committee, University of York. The animals were killed according to Schedule 1 of the UK Home Office Animals (Scientific Procedures) Act. Neurons were cultured in Neurobasal medium (Invitrogen) containing 2% B27 (Invitrogen), 5% foetal calf serum (PAA Lab, Pasching, Austria), 1 mM l‐glutamine, 35 mM glucose, 100 units/mL penicillin, and 0.1 mg/mL streptomycin (Invitrogen). Cytosine arabinoside (2.4 μM) was added to the cultures 2–4 days after plating to inhibit the proliferation of non‐neuronal cells. Hippocampal neurons were transfected with Lipofectamine 2000 according to the manufacturer's instructions (Invitrogen).

### Protein purification

Recombinant His and GST fusion proteins were expressed and purified according to a previously described protocol (Evans *et al*. 2001). When necessary, GST was removed from fusion proteins by incubation with PreScission protease (GE Healthcare) at 4°C overnight.

### *In vitro* phosphorylation

Phosphorylation reactions were prepared in kinase reaction buffer (100 mM Tris‐HCl pH 7.2, 25 mM MgCl_2_, 2 mM EGTA, 2 mM dithiothreitol, 250 μM NaVO_4_^3−^ and 5 mM MnCl_2_). Reactions were initiated by the addition of pre‐warmed ATP (250 μM final concentration) to 10 μg protein substrate and 100 nM C‐Src, incubated at 30°C for 3 h and terminated by the addition of 2× SDS sample buffer (Sigma).

### Mass spectrometry

*In vitro* untagged C‐Src‐phosphorylated Mint(1‐314) was separated from the other assay components of a similar mass [Src kinase and bovine serum albumin (BSA)] by 2D gel electrophoresis using a first dimension of pH 3–10. The Coomassie‐stained Mint protein spot was sampled in four locations, each with increasing mass and decreasing pI. Following sequential tryptic and chymotryptic digestion, peptides were subjected to reversed phase LC‐MS/MS using a Dionex Ultimate HPLC with a polystyrene–divinylbenzene monolithic column coupled to either an Applied Biosystems QSTAR Pulsar I or Bruker HCT ultra‐mass spectrometer. Collision‐induced dissociation mass spectra were searched against a small custom database containing the Mint1 sequence. Phosphorylation sites were assigned using Mascot scores. ‘Delta score’ differences (Savitski *et al*. 2011) and manual inspection of spectra were used to resolve ambiguities in site assignments.

### Immunoprecipitation

Transfected COS‐7 cells grown in 10 cm plates (10^7^ cells/plate) were lysed in ice‐cold radioimmunoprecipitation assay buffer(50 mM Tris pH 8.0, 150 mM NaCl, 1% TritonX‐100, 0.5% deoxycholate, 1 mM EDTA, 1 mM NaVO_4_^3^) and incubated overnight at 4°C with 25 μL Protein G resin (Genscript) and 3 μg of the appropriate antibody. Beads were washed three times in radioimmunoprecipitation assay buffer and proteins eluted in SDS sample buffer and processed for western blotting.

### Immunofluorescence

COS‐7, HeLa cells or hippocampal neurons were cultured on No. 1 glass coverslips (coated with poly d‐lysine for neurons) prior to transfection. Culture media containing 1 μg/mL doxycycline was applied to Flp‐in T‐REx HeLa cells to induce expression of APP‐FLAG for the indicated times. Cells were fixed in 4% paraformaldehyde with 4% sucrose and then permeabilised for 30 min in phosphate‐buffered saline (PBS) with 1% BSA and 0.1% Triton X‐100. Primary antibody diluted in PBS with 1% BSA was applied for 1 h at 25°C. After three washes in PBS, Alexa‐fluor‐conjugated secondary antibodies (1 : 500) were incubated for 30 min. Following three further washes in PBS, coverslips were mounted in Mowial mountant. Images were captured using a Zeiss LSM 710 Meta confocal microscope in the Technology Facility, Department of Biology, University of York or a Nikon TE200 epifluorescence microscope equipped with a Rolera‐XR CCD camera (QImaging, Surrey, BC, Canada).

### Data analysis

Densitometric analysis of protein bands on western blots and quantification of particle number in COS‐7 and HeLa immunofluorescent images was performed using ImageJ software (Abramoff *et al*. 2004). Neurite analysis of Mint‐ and APP‐transfected hippocampal neurons employed the NeuronJ plugin for ImageJ (Meijering *et al*. 2004). Where appropriate, statistical analysis of the data was performed with SigmaPlot (Systat, San Jose, CA, USA) software using one‐way anova with post hoc pair‐wise comparisons by Holm–Sidak tests. Chi‐squared analysis was used to test the statistical significance of cell phenotype data. A Bonferroni correction was used to correct for multiple comparisons.

## Results

### The Mint1 N‐terminus is sequentially phosphorylated by C‐Src

The only functional domains previously defined in the variable N‐termini of Mints1–3 are the Munc18‐binding domain in Mints 1 and 2 and the CASK interaction domain in Mint1 (Fig. 1d). A search for further functional domains or motifs in the Mint N‐termini revealed a highly conserved canonical C‐Src phosphorylation site and SH2 ligand motif (Songyang *et al*. 1993), YEEI, unique to Mint1 at Y202 (using numbering of the mouse protein, Genbank accession NP_796008). Furthermore, the N‐terminus of Mint1 is tyrosine rich, suggesting the potential for multiple phosphorylation events by tyrosine kinases. *In vitro* kinase assays using recombinant C‐Src demonstrated tyrosine phosphorylation of the Mint1 N‐terminus (1‐314), comparatively weak phosphorylation of the equivalent sequence in Mint2, and no phosphorylation of Mint3 (Fig. 1a).

**Figure 2:**
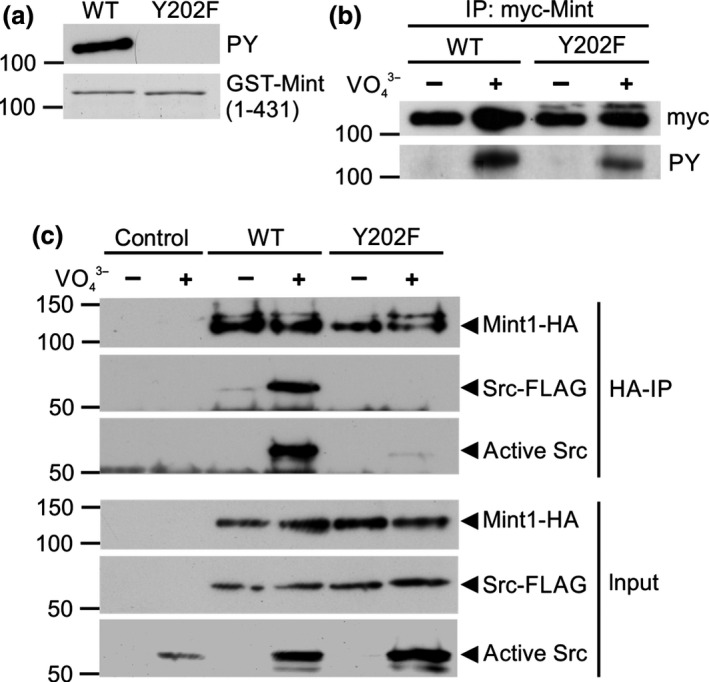
Mint1‐Y202 is phosphorylated in cells and recruits C‐Src kinase. (a) Recombinant GST Mint1(1‐431) protein and a Y202F mutant were subjected to the same phosphorylation assay described in Fig. 1a. The samples were processed for tyrosine phosphorylation (PY; top panel) and protein loading (Coomassie; bottom panel). (b) COS‐7 cells transfected with either wild‐type or mutant (Y202F) myc‐Mint1 N‐terminus (1‐431) were treated with or without NaVO_4_^3−^ prior to immunoprecipitation with anti‐myc antibodies. Immunoprecipitated Mint1 (top panel) and tyrosine phosphorylated Mint1 (bottom panel) were detected by immunoblotting. (c) COS‐7 cells transfected with C‐Src‐FLAG and either wild‐type or mutant (Y202F) Mint1‐HA N‐terminus (1‐431) were treated with or without NaVO_4_^3−^ prior to immunoprecipitation with anti‐HA antibodies. Input (bottom panel) or immunoprecipitated (top panel) Mint1, total C‐Src‐FLAG and active C‐Src phosphorylated on Y416 were detected by immunoblotting. All gels and immunoblots are representative of three independent experiments.

To investigate whether the predicted YEEI motif was indeed targeted by C‐Src, *in vitro* phosphorylated Mint1(1‐314) was resolved by 2D gel electrophoresis and the protein spot corresponding to tyrosine‐phosphorylated protein (as determined by western blotting; Fig. 1b) was analysed by LC‐MS/MS after digestion with trypsin and then chymotrypsin. The tryptic peptide 182‐AEDEPYAEPYADYGGLQEHVYEEIGDAPELEAR‐214 was observed in its mono‐, di‐ and tri‐phosphorylated forms, with phosphorylation of the YEEI motif at Y202 assigned in all three cases and subsequent phosphorylation of Y191 and Y187 in the di‐ and tri‐phosphorylated forms (Fig 1c). All forms were phosphorylated on Y202 and peptides containing intermediate phosphorylation of either Y187 or Y191 alone, or Y202 and Y187 together, were not observed. As expected, the stoichiometry of phosphorylation of Mint1(182‐214) was higher in 2D gel spots with lower apparent pI values, increasing from zero in spot 1 to three in spot 4 (Fig 1b). Assignments of mono‐ and di‐phosphorylated forms of the chymotryptic peptide 180‐HRAEDEPYAEPYADY‐194 indicated that Y194 was not phosphorylated. Thus, we concluded that Mint1 is sequentially phosphorylated by C‐Src in the order Y202, Y191 and Y187 as depicted in the schematic in Fig. 1d. To confirm the mass spectrometry data, we mutated the first predicted phosphorylation site, Y202, to phenylalanine and found that it completely abolished *in vitro* phosphorylation of Mint1(1‐431) by C‐Src (Fig. 2a).

### Mint1‐Y202 is tyrosine phosphorylated in cells and recruits active C‐Src to its N‐terminal domain

To assess whether C‐Src phosphorylates Mint1‐Y202 in cells, COS‐7 fibroblasts were transfected with myc‐Mint1(1‐431) wild‐type or a Y202F mutant. We found that in resting cells we could not detect phosphorylation of immunoprecipitated myc‐Mint1(1‐431) by immunoblotting with a phosphotyrosine antibody (Fig. 2b). However, activation of tyrosine kinase signalling with sodium pervanadate, an inhibitor of tyrosine phosphatases (Swarup *et al*. 1982), induced detectable tyrosine phosphorylation of Mint1(1‐431) that was reduced in the Y202F‐mutant protein (Fig. 2b).

In the light of the fact that Y202 is part of a canonical C‐Src‐SH2 ligand, YEEI, we examined the possibility that Mint1 is a scaffold for recruiting and activating C‐Src, potentially allowing the kinase to phosphorylate other proteins bound to Mint1. Lysates from COS‐7 cells transfected with Mint1(1‐431)‐HA and C‐Src‐FLAG were incubated in the presence or absence of pervanadate and then subjected to immunoprecipitation with anti‐HA antibodies and processed for western blotting. In agreement with the lack of resting phosphorylation of Mint1 (Fig. 2b) co‐immunoprecipitation of N‐terminal Mint1 with C‐Src was barely detectable (Fig. 2c). However, the binding was dramatically enhanced by treatment with pervanadate. Furthermore, C‐Src that was bound to Mint was active, as shown by detection of C‐Src auto‐phosphorylation on Y416 in the anti‐HA Mint1 immunoprecipitations. Mutation of Y202 in the Mint N‐terminus to phenylalanine‐abolished binding of active C‐Src (Fig. 2c), suggesting Y202 is the principle site for C‐Src recruitment to the Mint1 N‐terminus.

**Figure 3:**
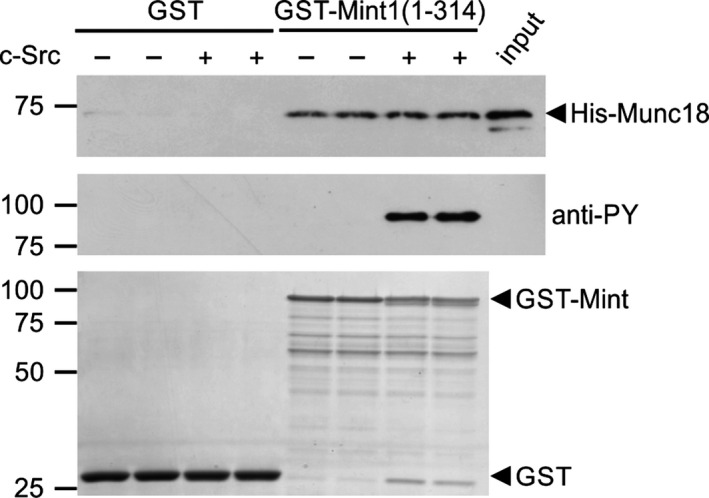
Tyrosine phosphorylation of Mint1 does not regulate Munc18 binding. GST or GST‐Mint1(1‐314) were incubated in duplicate in the presence or absence of C‐Src kinase prior to incubation with glutathione resin and bacterial lysate containing recombinant His‐Munc18. Following washing, Munc18 present in the bound fraction or in 0.5% of the input lysate was detected by immunoblotting (top panel). Phosphorylation of Mint1 was confirmed by anti‐phosphotyrosine (middle panel). Protein levels were determined by Coomassie staining of the PVDF membrane (bottom panel). The immunoblots are representative of three independent experiments.

The close proximity of Y202, Y187 and Y191 to the minimal sequence in Mint1 known to be sufficient for Munc18 binding (amino acids 226–314; Okamoto and Sudhof 1997) suggested that Mint1 tyrosine phosphorylation might regulate binding to Munc18. However, no significant effect of C‐Src phosphorylation on Mint1‐Munc18 binding was observed by *in vitro* GST pulldown assays (Fig. 3).

### Mint1‐Y202 regulates APP trafficking

**Figure 4:**
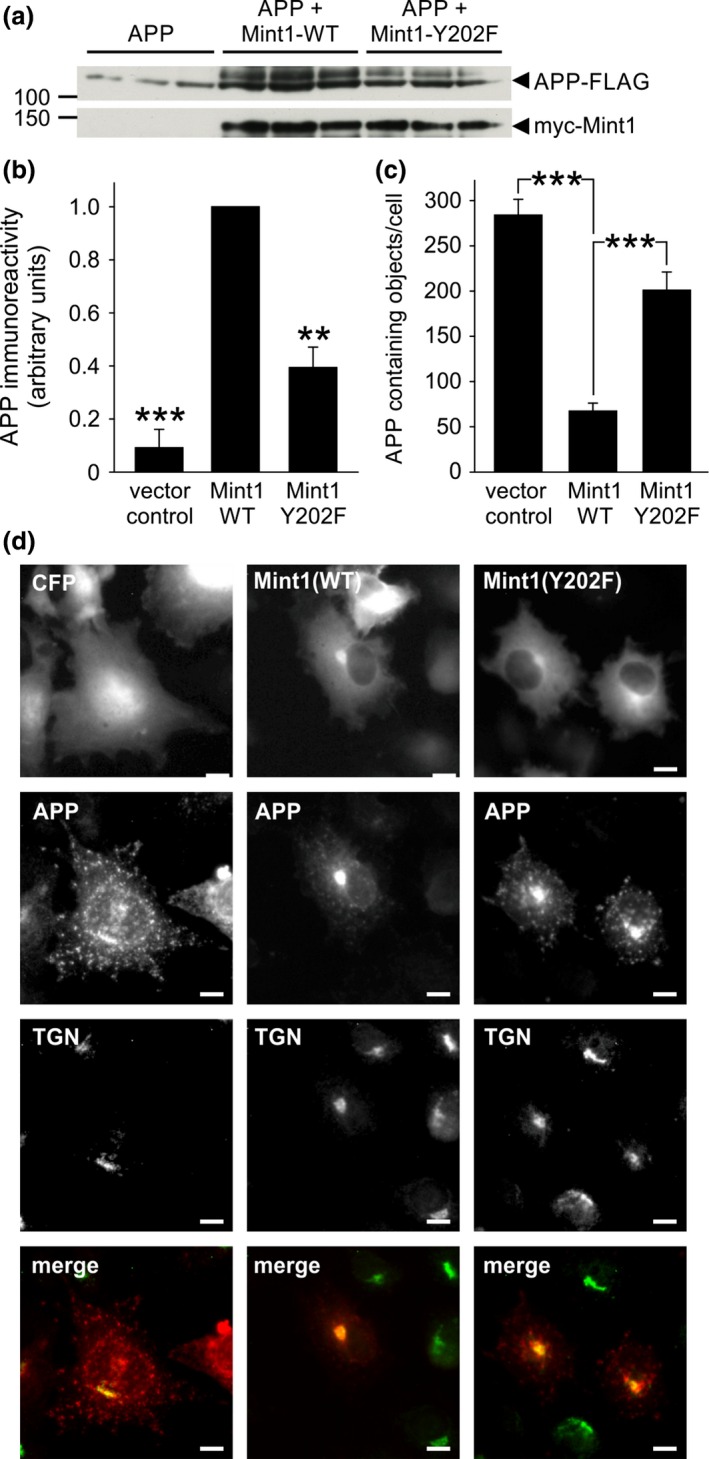
Mint1‐Y202 regulates amyloid precursor protein (APP) metabolism. (a) COS‐7 cells transfected in triplicate with APP‐FLAG and empty vector or myc‐Mint1‐WT or ‐Y202F were processed for immunoblotting with anti‐FLAG and anti‐myc antibodies. (b) APP immunoreactivity was quantified by densitometry and normalised to APP + Mint1‐WT. Data are plotted as mean ± SEM, *n* = 3 separate experiments, each performed in triplicate. **p < 0.01, ***p < 0.001. (c) COS‐7 cells transfected as described in (a) were subjected to immunocytochemistry with anti‐FLAG (APP) and anti‐myc (Mint1) antibodies and the number of FLAG reactive puncta/cell was counted using ImageJ software in myc positive cells. Data are from ≥ 20 cells per condition, ***p < 0.001. (d) Representative images are shown of COS‐7 cells transfected with APP‐FLAG and CFP or CFP‐Mint1‐WT or ‐Y202F that were stained with anti‐FLAG (red) and anti‐TGN‐46 (green) antibodies. Merged images are of the red and green channels only. Scale bar = 10 μm.

**Figure 5:**
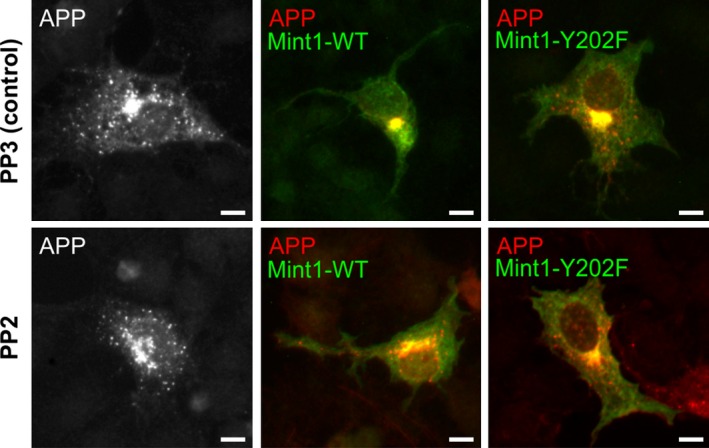
Regulation of amyloid precursor protein (APP) trafficking by Mint1 is Src dependent. COS‐7 cells were transfected with APP‐FLAG and empty myc vector (pMH) or myc‐Mint1‐WT or ‐Y202F and then incubated in the presence of 5 μM PP2 or its inactive analogue, PP3, for 48 h prior to immunocytochemistry with anti‐FLAG (APP; red) and anti‐myc (Mint1; green) antibodies. Representative images are shown of merged red and green channels except for APP alone which was co‐transfected with an empty vector. Scale bar = 10 μm.

We next considered the interaction of Mint1 with APP as a possible target for regulation by C‐Src phosphorylation. It has been shown that co‐expression of APP with Mint1 in heterologous cells results in a dramatic increase in the stability of APP that is hypothesised to be due to interaction of Mints and APP in the trans‐Golgi network (TGN; McLoughlin *et al*. 1999; Mueller *et al*. 2000; Biederer *et al*. 2002). In support, we also demonstrated that co‐expression of Mint1 increased the stability of APP as detected by western blotting (Fig 4a and b) and induced a perinuclear accumulation of Mint1 and APP that co‐localised with a TGN‐46 positive compartment (Fig 4d). Analysis of APP puncta in these cells by immunofluorescence demonstrated a reduced number of cytosolic APP puncta compared to APP expression alone (Fig. 4c). However, when Mint1‐Y202F was co‐expressed with APP there was a significant reduction in APP immunoreactivity and a significant increase in the number of APP puncta (Fig. 4c and d). To confirm that the effects of Y202 mutation on APP trafficking were because of an inhibition of Mint1 phosphorylation by Src, we repeated the co‐expression of APP and Mint in COS‐7 cells in the presence or absence of the Src family kinase inhibitor PP2 (Hanke *et al*. 1996) or its inactive analogue PP3 (Fig 5). PP3 had no effect on the localisation of APP puncta previously observed in Fig. 4. However, following incubation with PP2, despite perinuclear co‐localisation of Mint 1 and APP, there was an increase in cytosolic APP puncta, suggesting Src inhibition had partially reversed the perinuclear accumulation of APP by Mint1. PP2 had no effect on the Mint1‐Y202F‐dependent trafficking of APP, consistent with the point mutation inhibiting the effect of Src on Mint1 function and not acting as a general loss‐of‐function mutation.

**Figure 6:**
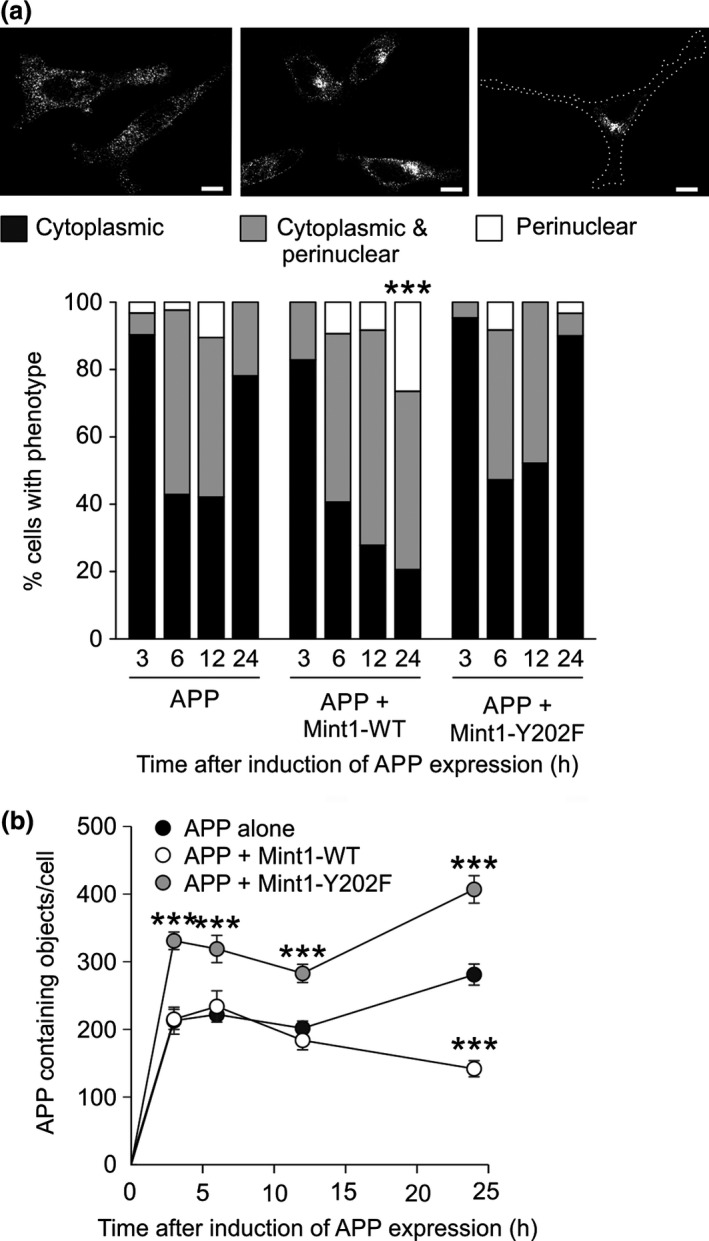
Amyloid precursor protein (APP) recycling is Mint1‐Y202 dependent. Tetracycline‐inducible APP‐FLAG Flp‐in HeLa cells were transfected with CFP, CFP‐Mint1‐WT or ‐Y202F and then induced to express APP‐FLAG for 3, 6, 12 or 24 h prior to processing for immunofluorescence with anti‐FLAG or anti‐CFP antibodies. (a) Top panels show representative grey scale images of cells with APP‐FLAG immunoreactivity predominantly cytoplasmic, perinuclear or distributed between the two (cytoplasmic and perinuclear). Cells from each time point were then classified into one of the three phenotypes according to the localisation of APP‐FLAG puncta. Data are plotted as percentages, *n* ≥ 30 cells for each condition. (b) The number of APP‐FLAG reactive puncta in each CFP positive cell was quantified using ImageJ software. Data are plotted as mean ± SEM, from ≥ 30 cells per condition. Pair‐wise chi‐squared statistical analysis was carried out for each condition, with Bonferroni correction. ***p < 0.0001, comparing all conditions at 24 h.

To investigate whether the accumulation of Mint1 was affecting Golgi exit of APP or a block in recycling, we generated a stable tetracycline‐inducible APP‐FLAG‐expressing flp‐in HeLa cell line in which we transfected wild‐type or Y202F‐mutant Mint. Twenty‐four hours after Mint1 transfection, FLAG‐APP expression was induced with doxycycline and the localisation and number of APP puncta was monitored over time (Fig. 6a). Intracellular APP puncta were detectable from 3 h post‐induction, with no statistical difference in APP localisation between conditions at 3, 6 and 12 h (All *p* values > 0.1, α value = 0.0166). Twenty‐four hours after APP induction, cells expressing Mint1‐Y202F and APP alone had a similar APP distribution to 3 h, with 90% and 78% of APP, respectively, being cytoplasmic. In contrast, 26% of cells expressing wild‐type Mint1 had large perinuclear puncta (Fig 6a; *p* < 0.0001 compared with other conditions) and the total number of APP puncta concomitantly decreased (Fig 6b). Conversely, Mint1‐Y202F transfection caused an increase in APP puncta at all time points (Fig 6b). We concluded that Mint1 is unlikely to affect the initial exit of APP from the Golgi but regulates an aspect of APP recycling and that this activity is dependent on Y202.

### Mint1‐Y202 alters the localisation of APP in neurons

**Figure 7:**
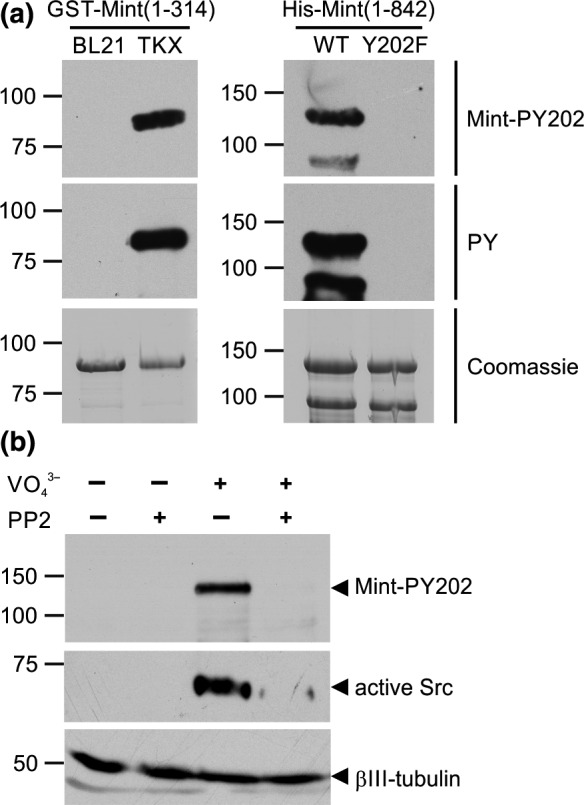
Mint1 is phosphorylated on Y202 in cultured neurons. (a) A phosphospecific antibody (anti‐Mint‐PY202) raised to a peptide containing phospho‐Y202 was tested for specificity on lysates of recombinant N‐terminal Mint1 expressed in TKX bacteria (left panel) and full length Mint1‐WT and ‐Y202F phosphorylated *in vitro* by C‐Src (right panel). The lower band of ~ 90 kDa in the right panels represents a proteolytic fragment of full length Mint1. (b) Lysates from cortical neurons incubated in the presence or absence of 100 μM sodium pervanadate and/or 10 μM PP2 were processed for immunoblotting with anti‐Mint1‐pY202, anti‐Src‐pY416 (active Src) or anti‐βIII‐tubulin.

To detect phosphorylation of endogenous Mint1 in neurons, we generated an affinity‐purified phosphospecific antibody to a 12mer peptide of the Mint sequence centred on phospho‐Y202 (as described in Materials and methods section). The antibody detected phosphorylation of recombinant Mint1 N‐terminus expressed in tyrosine kinase expressing TKX bacteria (Fig. 7a) and *in vitro* phosphorylation of full length His‐Mint1‐WT but not His‐Mint1‐Y202F (Fig. 7a). As observed in COS‐7 cells, the antibody did not detect Y202 phosphorylation under resting conditions, but revealed Mint1 phosphorylation in cortical neurons in which tyrosine kinases were activated by treatment with sodium pervanadate (Fig. 7b). The phosphorylation of Mint1 and Src kinases was abolished by pre‐treatment with the Src family kinase inhibitor PP2 (Fig 7b).

**Figure 8:**
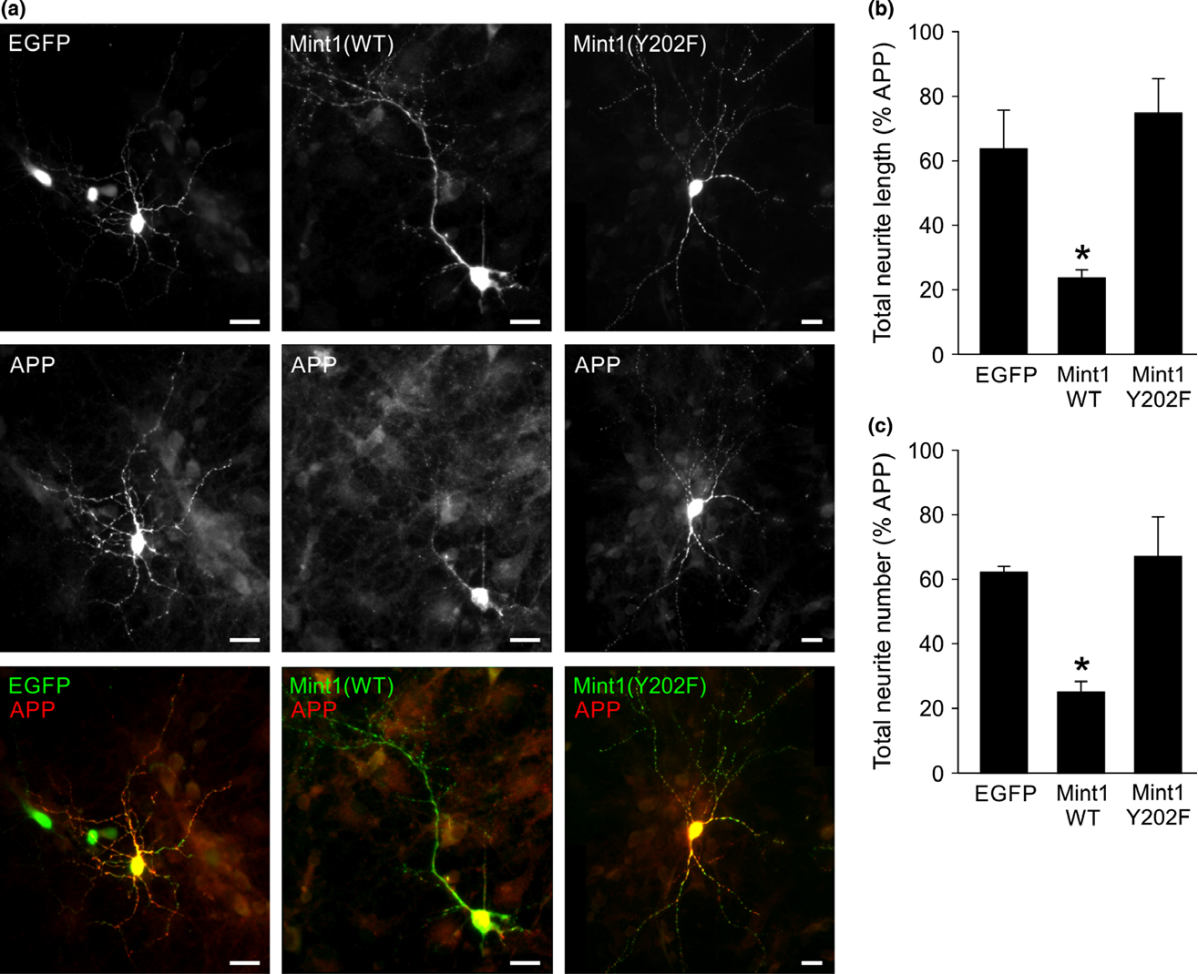
Mint1‐Y202 controls neuronal trafficking of amyloid precursor protein (APP). (a) Representative fluorescence images of cultured hippocampal neurons transfected with EGFP, myc‐Mint‐WT or myc‐Mint‐Y202F (top panels, grey scale) and APP‐FLAG (middle panels, grey scale). Bottom panels show merge of red and green channels. The extent of APP trafficking was quantified by calculating the neurite length (b) or neurite number (c) occupied by APP staining as a percentage of that occupied by EGFP or Mint staining. Data are mean ± SEM, *n* = 3 individual experiments (10–15 neurons analysed/condition/experiment). *p < 0.05 compared to EGFP or Mint‐Y202F. Scale bar = 20 μm.

To discover whether Mint1‐Y202 regulates APP trafficking in neurons, the co‐expression of APP and Mint1 plasmids was replicated in cultured hippocampal neurons. Here the effect was more striking as co‐expression of wild‐type Mint1 prevented APP from entering neurites and caused accumulation in the cell soma (Fig. 8a). Again, this phenotype was rescued when APP was co‐expressed with Mint1‐Y202F. We quantified the morphology of neurites stained for APP and Mint1 and found significant inhibition by wild‐type Mint1, but not Mint1‐Y202F, of the number and total length of neurites occupied by APP (Fig. 8b and c). Therefore the Mint1‐dependent reduction in APP trafficking is not just limited by its distance from the cell soma, but also its ability to traffic into certain processes.

## Discussion

We have discovered a novel sequential tyrosine phosphorylation of the Mint1 N‐terminus by C‐Src kinase. The specific downstream consequences of these phosphorylation events are the recruitment of active C‐Src and the regulation of APP sorting at the TGN. Our data identify Y202 as a determinant for Mint1‐dependent APP accumulation in the TGN and could have implications for the pathological trafficking of APP in AD.

Phosphorylation of the Mint1 N‐terminus by C‐Src *in vitro* was only detected on Y202, Y191 and Y187 by mass spectrometry. Other tyrosine residues in Mint1 have been shown to be phosphorylated in brain samples in phosphoproteomic screens (Wiśniewski *et al*. 2010), but mutation of Y202 completely abrogated C‐Src binding and hence we propose that Y202, Y191 and Y187 are the only N‐terminal C‐Src phosphorylation sites *in vivo*. The residual tyrosine phosphorylation of Mint1(1‐431)‐Y202F in vanadate treated COS‐7 cells (Fig. 2b) is likely due to phosphorylation by other tyrosine kinases. An explanation for why Y202 phosphorylation has not been previously detected in the brain by phosphoproteomic screens is that such studies are often conducted on resting cells or tissue. It is likely that under these conditions, the stoichiometry of phosphorylation at Y202 is low, similar to what we have observed in heterologous cells and cultured neurons. In support, the only references to Mint1 Y202 phosphorylation that appear in the PhosphoSitePlus database refer to in‐house‐curated data sets from Jurkat cells treated with the phosphatase inhibitors pervanadate and calyculin A (Hornbeck *et al*. 2012). Therefore, phosphorylation of Y202 cycles rapidly and/or is only increased when C‐Src is strongly activated.

The principal effects of preventing tyrosine phosphorylation of Mint1 were a decrease in the accumulation of APP in the TGN and enhanced transport of APP along neurites in neurons. Using inducible expression of APP, we demonstrated that the accumulation of APP likely arises after its endocytosis rather than prior to its initial secretion. Following internalisation, APP is sorted from early endosomes either to the TGN by the retromer complex or to the late endo‐lysosomal system (Vieira *et al*. 2010; Bhalla *et al*. 2012; Choy *et al*. 2012). Thus, the accumulation of APP in the TGN by co‐expression of wild‐type Mint1 with APP could be due to the exclusive sorting of all internalised APP by the retromer complex and/or an inhibition of APP exit from the TGN. Conversely, Mint(Y202F) that cannot be phosphorylated favours exit from the TGN and permits axonal transport of APP. This supports the idea that in healthy neurons, transient turnover of C‐Src phosphorylation of Mint1 slows APP trafficking in the TGN. A similar function has been attributed to the APP sorting receptor and AD risk factor, SORL1, whose over‐expression also leads to the perinuclear accumulation of APP and an inhibition of its transport in neurites (Schmidt *et al*. 2007; Fjorback *et al*. 2012). It is hypothesised that SORL1‐dependent retention of APP in the TGN reduces its amyloidogenic processing and β‐amyloid peptide secretion (Willnow and Andersen 2013). It would be interesting to investigate whether Mint1 and/or Src interacts with or regulates SORL1.

We observed very weak phosphorylation of the Mint2 N‐terminus and no phosphorylation of the Mint3 N‐terminus in our *in vitro* kinase assay using recombinant C‐Src. The lack of Mint3 tyrosine phosphorylation suggests Src phosphorylation of Mints 1 and 2 is a neuronal adaptation of its function and hence consistent with its role in neuronal APP trafficking. A previous study focussing on Mint2 tyrosine phosphorylation, also found the extent of Mint1 phosphorylation by C‐Src *in vitro* and in cells was many fold higher than that of Mint2 (Chaufty *et al*. 2012). Y202 is not conserved in the Mint2 sequence suggesting the tyrosine phosphorylation we have characterised is another difference between these isoforms in addition to the CASK‐binding site identified in the Mint1 N‐terminus (Borg *et al*. 1998). Furthermore, preventing Mint2 tyrosine phosphorylation by Src had a different effect on APP trafficking to what we observed for Mint1. An un‐phosphorylatable mutant of Mint2 (Y86,110,193F) caused Mint2 to accumulate in the Golgi, but promoted APP recycling (Chaufty *et al*. 2012). In further contrast to Mint1, wild‐type Mint2 was found to have no effect on the stability of APP or its perinuclear accumulation (Chaufty *et al*. 2012). Thus, the N‐terminal phosphorylation of Mints 1 and 2 has the potential to differentially regulate APP trafficking.

How might multisite tyrosine phosphorylation of Mint1 by C‐Src regulate APP at the molecular level? First, Mint1 phosphorylation could allosterically regulate the interaction between its PTB domain and the APP C‐terminus. There are conflicting data regarding this mode of regulation, for example, deletion of the Mint1 N‐terminus inhibits the effect of the full length protein on APP processing (Mueller *et al*. 2000) and Sakuma *et al*. (2009) showed that stress‐induced serine phosphorylation of the Mint1/2 N‐terminus reduces APP binding. However, it was shown that tyrosine phosphorylation of Mint2 regulates APP trafficking without altering binding to APP (Chaufty *et al*. 2012). It is also possible that wild‐type Mint1 is acting via secondary interactions that are mediated by tyrosine phosphorylation. Surprisingly, we did not see phosphorylation‐dependent binding to Munc18 despite Y202, Y191 and Y187 being adjacent to the Munc18‐binding domain of Mint1 (Okamoto and Sudhof 1997). In addition to the recruitment of C‐Src itself, the three phosphorylated tyrosine residues of Mint1 might act as SH2 ligands for other signalling proteins, as observed in other multisite substrates of Src, such as p130^cas^ (Cary *et al*. 1998). Identification of the phosphospecific binding partners of Mint1 could help explain the radically different APP localisation we observe between the wild‐type and Y202F forms of Mint1 in neurons.

While the signalling adaptors Shc and Grb2 are recruited to the tyrosine‐phosphorylated tail of APP (Tarr *et al*. 2002; Zhou *et al*. 2004), the binding of the three major PTB domain‐containing APP adaptors, Dab1, Fe65 and Mint, are unaffected by APP tyrosine phosphorylation (Ando *et al*. 2001; Tamayev *et al*. 2009). However, Dab1 interacts with Fyn and Fe65 interacts with Abl, to regulate APP at the cell surface (Zambrano *et al*. 2001; Hoe *et al*. 2008), and we now show that Mint1 can interact with C‐Src to alter the trafficking of APP at the TGN. Taken together, PTB‐containing adaptors act as scaffolds to recruit and activate non‐receptor tyrosine kinases to orchestrate the different stages of APP trafficking.
